# Hepatitis B Virus e Antigen Activates the Suppressor of Cytokine Signaling 2 to Repress Interferon Action

**DOI:** 10.1038/s41598-017-01773-6

**Published:** 2017-05-11

**Authors:** Yi Yu, Pin Wan, Yanhua Cao, Wei Zhang, Junbo Chen, Li Tan, Yan Wang, Zhichen Sun, Qi Zhang, Yushun Wan, Ying Zhu, Fang Liu, Kailang Wu, Yingle Liu, Jianguo Wu

**Affiliations:** 1 State Key Laboratory of Virology and College of Life Sciences, Wuhan University, Wuhan, 430071 China; 2 Life Sciences Institute, Zhejiang University, Hangzhou, China

## Abstract

Hepatitis B virus (HBV) infection causes acute hepatitis B (AHB), chronic hepatitis B (CHB), liver cirrhosis (LC), and eventually hepatocellular carcinoma (HCC). The presence of hepatitis B e antigen (HBeAg) in the serum generally indicates ongoing viral replication and disease progression. However, the mechanism by which HBeAg regulates HBV infection remains unclear. Interferons (IFNs) are pleiotropic cytokines that participate in host innate immunity. After binding to receptors, IFNs activate the JAK/STAT pathway to stimulate expression of IFN-stimulated genes (ISGs), leading to induction of antiviral responses. Here, we revealed that HBeAg represses IFN/JAK/STAT signaling to facilitate HBV replication. Initially, HBeAg stimulates the expression of suppressor of cytokine signaling 2 (SOCS2). Subsequently, SOCS2 impairs IFN/JAK/STAT signaling through reducing the stability of tyrosine kinase 2 (TYK2), downregulating the expression of type I and III IFN receptors, attenuating the phosphorylation and nucleus translocation of STAT1. Finally, SOCS2 inhibits the expression of ISGs, which leads to the repression of IFN action and facilitation of viral replication. These results demonstrate an important role of HBeAg in the regulation of IFN action, and provide a possible molecular mechanism by which HBV resists the IFN therapy and maintains persistent infection.

## Introduction

HBV infection is a major cause of chronic hepatitis, liver cirrhosis, and hepatocellular carcinoma (HCC)^1, 2^. Although the mechanism of HBV pathogenesis remains largely unknown, it is believed that immune responses are the major causes of HBV pathogenesis^3^. Interferons (IFNs) are key cytokines that play important roles in innate immunity and antiviral responses^4^. Type I IFNs possess strong intrinsic antiviral activity through binding to the receptors, IFN-α/β receptor 1 (IFN-α/βR1) and IFN-α/β receptor 2 (IFN-α/βR2)^5, 6^. Type III IFNs, including IFN-λ1, IFN-λ2, and IFN-λ3, bind to a novel receptor composing cytokine receptor family 2 member 12 (CRF2-12 or IFN-λR1) and cytokine receptor family 2 member 4 (CRF2-4 or IL-10Rβ)^7^. Although type I and type III IFNs recognize different receptors, they share many common biological activities^8, 9^. After binding to receptors, IFNs activate JAK/STAT pathway^10^. Activated STAT1/2 are heterodimerized and interact with IFN regulatory factor 9 (IRF-9) to form ISG factor 3 (ISGF3), which subsequently translocates into nucleus and binds to IFN-stimulated response element (ISRE) on IFN-stimulated genes (ISGs), leading to the expression of ISGs^11^. IFN-α inhibits HBV replication in a variety of systems and is used therapeutically to treat HBV infection. However, about 70% of CHB patients respond poorly to exogenous IFN-α treatment^12–14^. Thus, HBV must develop strategies to counteract IFN action, which may contribute to the ineffectiveness of IFN-α therapy^15, 16^.

Although hepatitis B e antigen (HBeAg) is not required for HBV replication and its exact function is unclear, it may play a role in chronic HBV infection as HBeAg in the serum generally indicates ongoing HBV replication and liver disease^17–19^. The emergence of HBeAg-negative variants correlates with an exacerbation of liver injury and even with viral clearance in some patients^20, 21^. HBeAg is responsible for modulation of host immune response during CHB progression, inhibits TLR-2 expression, and abrogates the antiviral activity of TLR signaling *via* suppressing IFN-β and ISG production^22–25^. Despite the important clinical implications, the function of HBeAg in IFN action and the molecular mechanism by which HBeAg regulates IFN remains largely unknown.

Members of the intracellular suppressor of cytokine signaling (SOCS) family are regulators of cytokine signaling pathways^26, 27^. Eight members (SOCS1 to 7 and CIS) are identified, and most SOCSs are induced by cytokines and act in a classical negative-feedback loop to inhibit cytokine signaling^28^. Most SOCS proteins are induced by cytokines and act in a classical negative-feedback loop to inhibit cytokine signaling. SOCS1 and SOCS3 inhibit interferon-mediated antiviral and antiproliferative activities, and are upregulated in brain resident cells in response to virus-induced inflammation of the central nervous system *via* at least two distinctive pathways^29, 30^.

Here, we investigated the mechanism by which HBV resists to IFN action and maintains persistent infection. Our results revealed that HBeAg initially activates SOCS2 through ERK pathway. HBeAg-activated SOCS2 subsequently reduces tyrosine kinase 2 (TYK2) stability, down-regulates IFN receptors expression, represses STAT1 phosphorylation, and finally attenuates ISGs production. Thus, we revealed a novel mechanism by which HBeAg and SOCS2 are coordinated to enhance HBV replication by hijacking the IFN/JAK/STAT pathway and attenuating IFN antiviral action.

## Results

### HBeAg attenuates STAT1 phosphorylation and nuclear translocation

**Figure 1:**
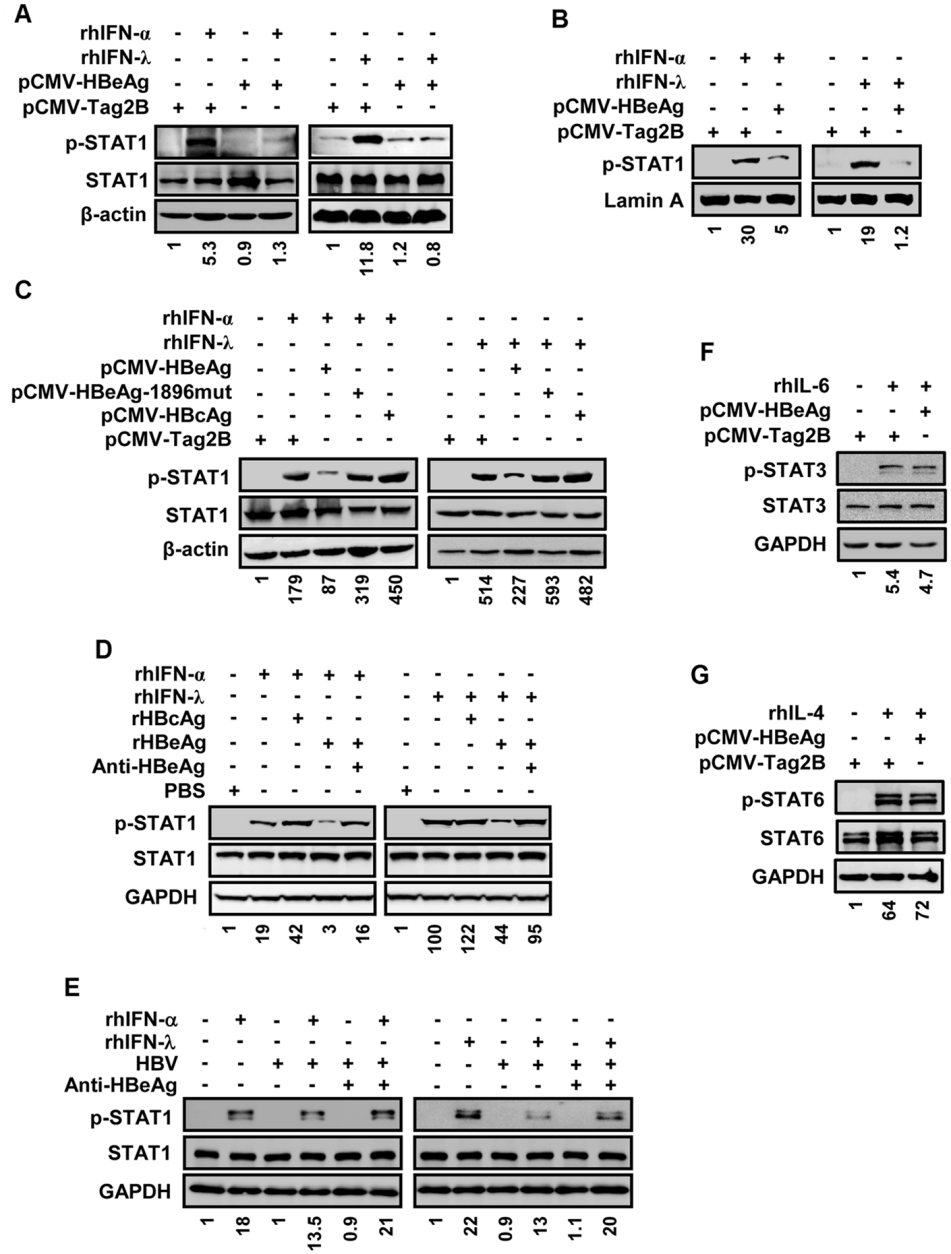
The effect of HBeAg on phosphorylation and nuclear translocation of STAT1 induced by IFN-α and IFN-λ1. (**A** and **B**) HepG2 cells were transfected with pCMV-Tag2B or pCMV-HBeAg for 48 h and then treated with recombinant human IFN-α (rhIFN-α) at 300 U/ml or recombinant human IFN-λ1 (rhIFN-λ1) at 100 ng/ml for 30 min. Cells were harvested and lysed, and p-STAT1, STAT1, and β-actin proteins in the cell lysates were detected by Western blot analyses (**A**). Nuclear extracts were prepared from the treated cells, and proteins in nuclear extracts were examined by Western blot analyses using anti-p-STAT1 antibody and anti-Lamin A antibody (**B**). (**C**) HepG2 cells were transfected with pCMV-Tag2B, pCMV-HBeAg, pCMV-HBeAg-1896mut, or pCMV-HBcAg for 48 h and then treated with rhIFN-α at 300 U/ml or rhIFN-λ1 at 100 ng/ml for 30 min. Cells were harvested and lysed, and p-STAT1, STAT1, and β-actin proteins in the cell lysates were detected by Western blot analyses. (**D**) HepG2 cells were pretreated with or without anti-HBeAg (10 μg/ml) for 12 h, and then incubated with PBS, recombinant hepatitis B c antigen (rHBcAg) or recombinant HBeAg (rHBeAg) at 50 ng/ml for 24 h and treated with rhIFN-α at 300 U/ml or rhIFN-λ1 at 100 ng/ml for another 30 min. Cells were harvested and lysed, and p-STAT1, STAT1, and GAPDH proteins in the cell lysates were detected by Western blot analyses. (**E**) HepG2-NTCP cells were mock infected or infected with HBV at 1,000 GEq per cell for 3 days, and then incubated with or without anti-HBeAg (10 μg/ml) for 36 h and treated with rhIFN-α at 300 U/ml or rhIFN-λ1 at 100 ng/ml for another 2 h. Cells were harvested and lysed, and p-STAT1, STAT1, and GAPDH proteins in the cell lysates were detected by Western blot analyses. (**F**) HepG2 cells were transfected with pCMV-Tag2B or pCMV-HBeAg for 48 h and treated with recombinant human IL-6 (rhIL-6) at 40 ng/ml for 30 min. Cells were harvested and lysed, and p-STAT3, STAT3, and GAPDH proteins in the cell lysates were detected by Western blot analyses. (**G**) HepG2 cells were transfected with pCMV-Tag2B or pCMV-HBeAg for 48 h and treated with recombinant human IL-4 protein (rhIL-4) at 20 ng/ml for 30 min. Cells were harvested and lysed, and p-STAT6, STAT6, and GAPDH proteins in the cell lysates were detected by Western blot analyses.

We initially evaluated the role of HBeAg in the phosphorylation of STAT1 induced by IFN in cells transfected with pCMV-HBeAg or pCMV-Tag2B and treated with recombinant human IFN-α (rhIFN-α) or recombinant human IFN-λ (rhIFN-λ). HBeAg was highly expressed in pCMV-HBeAg transfected cells and mainly secreted to the cell culture supernatant (Fig. S1A). Phosphorylation of STAT1 (p-STAT1) was enhanced by rhIFN-α or rhIFN-λ1 but repressed by HBeAg (Fig. 1A), and p-STAT1 in nucleus was enhanced by rhIFN-α or rhIFN-λ1 but reduced by HBeAg (Fig. 1B), suggesting that HBeAg plays an inhibitory role in IFN-induced phosphorylation and nuclear translocation of STAT1.

During HBV infection, seroconversion from HBeAg to anti-HBe may lead to the emergence of replication-competent HBV mutants that are unable to secrete HBeAg, and the most frequent mutation is a guanine (G)-to-adenine (A) change at nt 1896^31, 32^. Thus, we evaluated the indispensable role of HBeAg in the activation of STAT1 using HBeAg-1896mut. Cells were transfected with pCMV-Tag2B, pCMV-HBeAg, pCMV-HBeAg-1896mut, and pCMV-HBcAg (expressing hepatitis B core antigen), respectively, and treated with rhIFN-α or rhIFN-λ1. HBeAg was secreted in the cells transfected with pCMV-HBeAg, but not in the cells transfected with pCMV-HBeAg-1896mut or pCMV-HBcAg (Fig. S1B). Phosphorylation of STAT1 was induced by rhIFN-α or rhIFN-λ1 and such activation was repressed by HBeAg, but not affected by HBeAg-1896mut or HBcAg (Fig. 1C). The role of HBeAg in the regulation of STAT1 was also determined using a recombinant HBeAg (rHBeAg). The levels of rHBeAg protein in the cell culture medium were evaluated by ELISA (Fig. S1C). Cells were then treated with anti-HBeAg antibody, incubated with rHBeAg or rHBcAg, and treated with rhIFN-α or rhIFN-λ1. Similarly, p-STAT1 was stimulated by rhIFN-α or rhIFN-λ1 and such activation was repressed by rHBeAg but not by rHBcAg (Fig. 1D). More interestingly, the suppression of p-STAT1 mediated by HBeAg was rescued by anti-HBeAg (Fig. 1D). These results suggested that secreted HBeAg rather than intracellular HBeAg is more relevant for the repression of STAT1.

More importantly, we further confirmed the role of HBeAg in the regulation of STAT1 in a HBV infection context. HBeAg and HBsAg proteins can be detected in the cell culture supenatants 3 days after infection using ELISA, which demonstrated that HepG2-NTCP cells were successfully infected by HBV (Fig. S1D). p-STAT1 stimulated by rhIFN-α or rhIFN-λ1 was decreased in HBV-infected HepG2-NTCP cells than that in mock infected cells. And such suppression of p-STAT1 by HBV was nearly reversed by anti-HBeAg (Fig. 1E). These results demonstrated not only an inhibitory effect of HBeAg on STAT1 activation in an infection system, but also an indispensable role of HBeAg in HBV-mediated antagonism of IFN action.

Furthermore, the specificity of HBeAg inhibitory effect on STAT1 was confirmed. Cells were transfected with pCMV-Tag2B or pCMV-HBeAg and treated with recombinant human IL-6 (rhIL-6) or recombinant human IL-4 (rhIL-4). p-STAT3 was activated by rhIL-6 (Fig. 1F) and p-STAT6 was activated by rhIL-4 (Fig. 1G), but such activations were not affected by HBeAg. Taken together, we demonstrated that HBeAg is involved in the regulation of IFN/JAK/STAT signaling by specifically inhibiting IFN-mediated phosphorylation and nuclear translocation of STAT1.

### HBeAg represses IFN receptor expression and TYK2 phosphorylation

**Figure 2:**
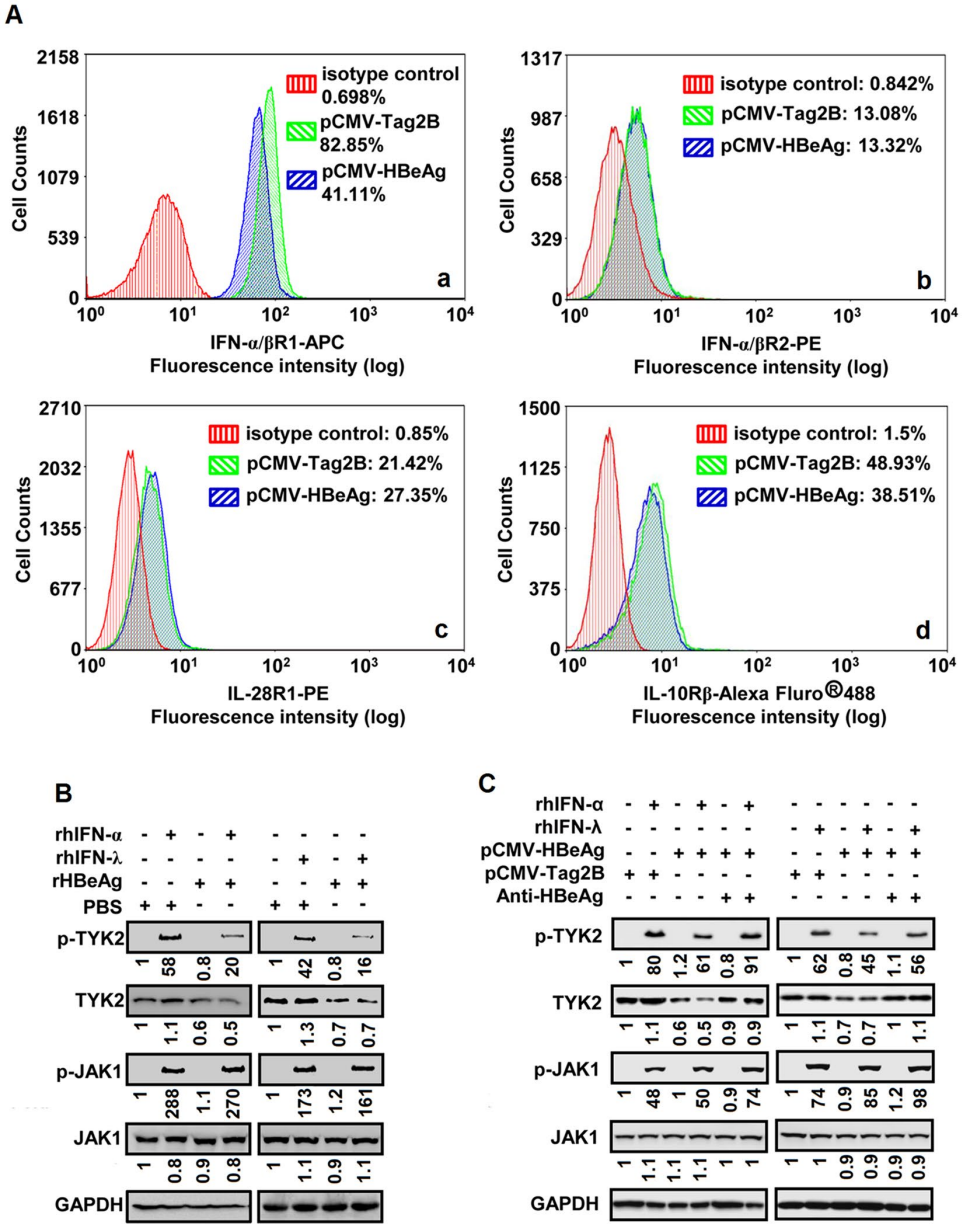
The role of HBeAg in regulation of IFN receptors expression, TYK2 stability and TYK2 phosphorylation induced by IFN-α and IFN-λ1. (**A**) HepG2 cells were transfected with pCMV-Tag2B or pCMV-HBeAg for 48 h. Cells were Fc-blocked by treatment with human IgG prior to staining. 5 × 10^5^ to 1 × 10^6^ cells in PBS buffer were incubated with specific antibody and analyzed by flow cytometry using a FACSCalibur (Beckman Coulter) to detect the levels of IFN-α/βR1 (a), IFN-α/βR2 (b), IL-28R1 (c), and IL-10Rβ (d) proteins. (**B**) HepG2 cells were incubated with PBS or rHBeAg at 50 ng/ml for 24 h, and treated with rhIFN-α at 300 U/ml or rhIFN-λ1 at 100 ng/ml for 30 min. Cells were harvested and lysed, and p-TYK2, TYK2, p-JAK1, JAK1, and GAPDH proteins in the cell lysates were detected by Western blot analyses. (**C**) HepG2 cells were transfected with pCMV-Tag2B or pCMV-HBeAg for 12 h, and incubated with anti-HBeAg (10 μg/ml) for another 36 h. Cells were treated with rhIFN-α at 300 U/ml or rhIFN-λ1 at 100 ng/ml for 30 min before harvest. Cells were lysed, and p-TYK2, TYK2, p-JAK1, JAK1, and GAPDH proteins in the cell lysates were detected by Western blot analyses.

We verified whether the inhibitory effect of HBeAg on STAT1 phosphorylation was caused by the dysregulation of IFN receptors. Initially, we showed that HBeAg was properly expressed in pCMV-HBeAg-transfected cells (Fig. S1A). The effects of HBeAg on the regulation of IFN-α receptors (IFN-α/βR1 and IFN-α/βR2) and IFN-λ1 receptors (IL-28R1 and IL-10Rβ) were determined by flow cytometry analyses. HBeAg significantly attenuated the production of IFN-α/βR1 (Fig. 2Aa) and IL-10Rβ (Fig. 2Ad), but not IFN-α/βR2 (Fig. 2Ab) and IL-28R1 (Fig. 2Ac). Similarly, Western blot analyses showed that HBeAg repressed the production of IFN-α/βR1 (IFNAR1) and IL-10Rβ, but not IFN-α/βR2 (IFNAR2) and IL-28R1 (Fig. S2B). These results suggested that HBeAg represses the activities of IFN-α and IFN-λ1 through attenuating their receptors.

IFN-α and IFN-λ1 activate JAK1 or TYK2 to regulate ISGs expression and immune response. Thus, we evaluated the effect of HBeAg on the regulation of JAK1 and TYK2 in cells treated with rHBeAg, rhIFN-α, and rhIFN-λ, respectively. The levels of HBeAg in the conditioned media were evaluated by ELISA (Fig. S2C). Interestingly, p-TYK2 was up-regulated by rhIFN-α or rhIFN-λ1 and down-regulated by rHBeAg, whereas p-JAK1 was stimulated by rhIFN-α or rhIFN-λ1 but not affected by rHBeAg (Fig. 2B). In addition, cells were transfected with pCMV-tag2B or pCMV-HBeAg, and then treated with rhIFN-α, rhIFN-λ, or anti-HBeAg. p-TYK2 was activated by rhIFN-α or rhIFN-λ, whereas such activation was repressed by HBeAg and restored by anti-HBeAg (Fig. 2C). p-JAK1 was also activated by rhIFN-α or rhIFN-λ, but this activation was not affected by HBeAg (Fig. 2C). We also noticed that extracellular adding of rHBeAg and transfection of pCMV-HBeAg could lead to the reduction of TYK2 protein (Fig. 2B and C). Taken together, we demonstrated that HBeAg attenuates IFN-α and IFN-λ1 actions by down-regulating IFN-α/βR1 and IL-10Rβ expression, repressing TYK2 activation, and inhibiting STAT1 phosphorylation.

### HBeAg attenuates IFN-induced expression of ISGs and anti-HBV activity

**Figure 3:**
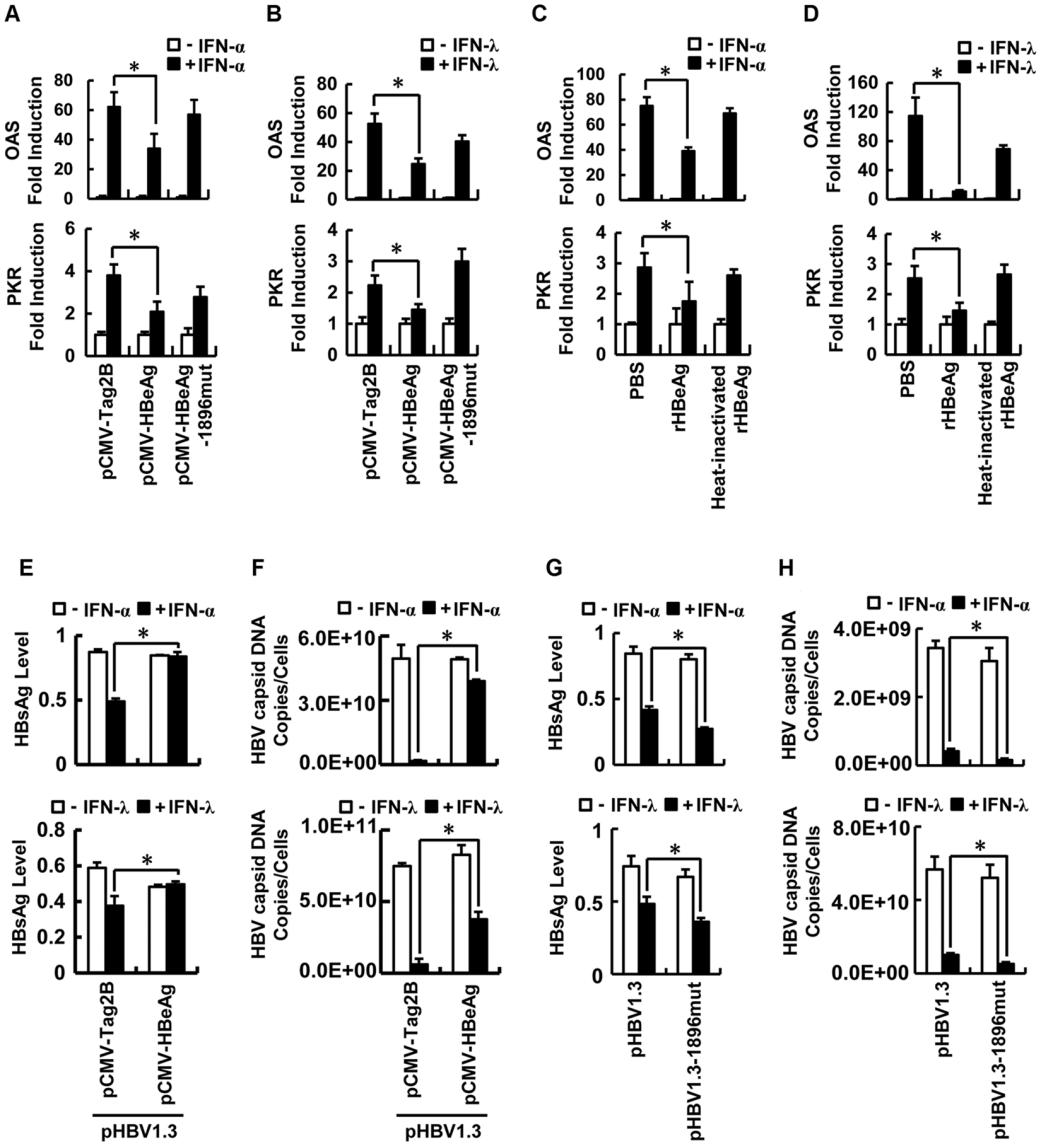
The impacts of HBeAg on ISG expression and antiviral activity induced by IFNs. (**A** and **B**) HepG2 cells were transfected with pCMV-Tag2B, pCMV-HBeAg, or pCMV-HBeAg-1896mut for 24 h and treated with rhIFN-α (**A**) or rhIFN-λ1 (**B**) for another 24 h. Total RNA was extracted from the treated cells, and mRNA levels of IFN-stimulated genes, OAS (upper panels) and PKR (lower panels), in the RNA extracts were detected by real-time PCR. (**C** and **D**) HepG2 cells were incubated with PBS, rHBeAg at 50 ng/ml, or heat-inactivated rHBeAg for 12 h and treated with rhIFN-α (**C**) or rhIFN-λ1 (**D**) for another 24 h. Total RNA was extracted from the treated cells, and mRNA levels of OAS (upper panels) and PKR (lower panels) in the RNA extracts were detected by real-time PCR. (**E** and **F**) HepG2 cells were co-transfected with pHBV1.3 and pCMV-Tag2B or pCMV-HBeAg for 24 h and treated with rhIFN-α (upper panels) or rhIFN-λ1 (lower panels) for another 24 h. Cells were harvested and lysed, and the levels of hepatitis B s antigen (HBsAg) in culture supernatants were measured by ELISA using an HBsAg diagnostic kit (**E**). Cells were homogenized in lyses buffer and treated with DNase. The viral cores were precipitated by adding EDTA and polyethylene glycol and concentrated by centrifugation. HBV capsid-associated DNA was quantified by real-time PCR (**F**). (**G** and **H**) Huh7 cells were transfected with pHBV1.3 or pHBV1.3-1896mut for 24 h, and treated with rhIFN-α (upper panels) or rhIFN-λ1 (lower panels) for another 24 h. Cells were harvested and lysed, and the levels of hepatitis B s antigen (HBsAg) in culture supernatants were measured by ELISA (**G**). Cells were homogenized in lyses buffer and treated with DNase. The viral cores were precipitated by adding EDTA and polyethylene glycol and concentrated by centrifugation. HBV capsid-associated DNA was quantified by real-time PCR (**H**). Data shown were means ± SE; n = 3. *p < 0.05.

Because HBeAg down-regulates the actions of IFN-α and IFN-λ1, we evaluated the effect of HBeAg on the expression of ISGs, dsRNA-activated protein kinase (PKR) and 2′,5′-oligoadenylate synthetase (OAS). Cells were transfected with pCMV-Tag2B, pCMV-HBeAg, or pCMV-HBeAg-1896mut and treated with rhIFN-α or rhIFN-λ1. OAS and PKR mRNAs were up-regulated by rhIFN-α and rhIFN-λ1, whereas such activations were repressed by HBeAg but not by HBeAg-1896mut (Fig. 3A and B). In addition, cells were incubated with PBS, rHBeAg, or heat-inactivated rHBeAg, and then treated with rhIFN-α or rhIFN-λ1. Similarly, OAS and PKR mRNAs were enhanced by rhIFN-α and rhIFN-λ1, whereas such regulations were repressed by rHBeAg but not by heat-inactivated rHBeAg (Fig. 3C and D). Thus, we revealed that HBeAg attenuates the expression of ISGs induced by IFN-α and IFN-λ.

The role of HBeAg in the attenuation of antiviral action of IFN-α and IFN-λ was further confirmed in cells co-transfected with pHBV1.3 and pCMV-Tag2B or pCMV-HBeAg and treated with rhIFN-α or rhIFN-λ1. The expression of HBeAg in the cell culture medium was verified by ELISA (Fig. S3A). Interestingly, HBsAg (Fig. 3E) and HBV capsid-associated DNA (Fig. 3F) were significantly repressed by rhIFN-α and rhIFN-λ1 in the absence of HBeAg, but slightly down-regulated by IFN-α or IFN-λ1 in the presence of HBeAg, revealing that HBeAg attenuates the antiviral activities of IFN-α and IFN-λ1. Furthermore, Huh7 cells were transfected with pHBV1.3 or pHBV1.3-1896mut^33^, and treated with rhIFN-α or rhIFN-λ1. HBeAg was detected in the culture medium of cells transfected with pHBV1.3, but not expressed in cells transfected with pHBV1.3-1896mut (Fig. S3B). HBsAg (Fig. 3G) and HBV capsid-associated DNA (Fig. 3H) were slightly down-regulated by IFN-α and IFN-λ1 in the presence of HBV1.3, but significantly attenuated by IFN-α and IFN-λ1 in the presence of HBV1.3-1896mut, indicating that HBeAg is required for the regulation of IFN-α and IFN-λ1. Taken together, we demonstrated that HBeAg attenuates the actions of IFN-α and IFN-λ1.

### HBeAg activates SOCS2 expression through ERK signaling

**Figure 4:**
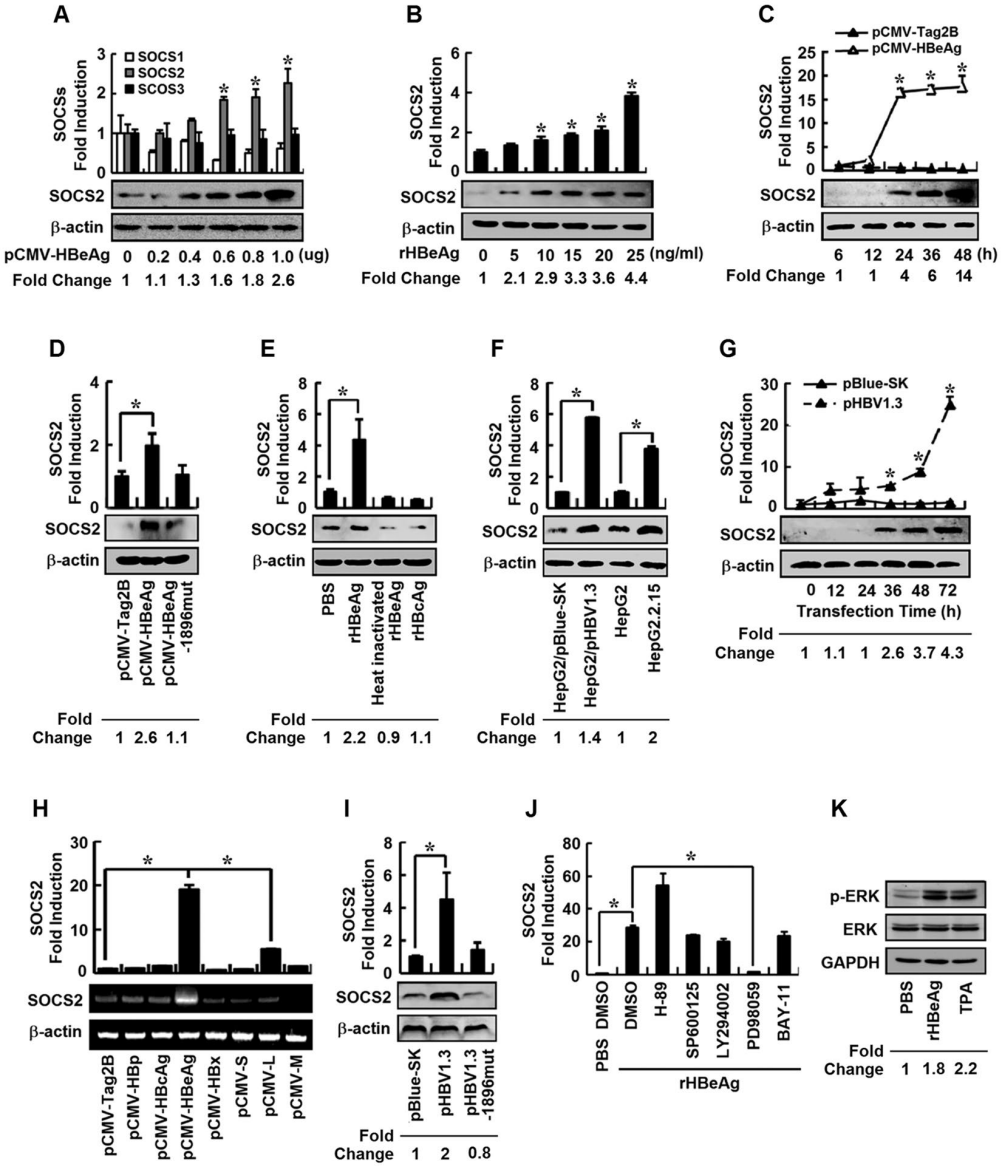
The effect of HBeAg on regulation of SOCS2 expression and ERK signaling. (**A**) HepG2 cells were transfected with pCMV-HBeAg at different concentrations for 24 h. SOCS1, SOCS2, and SOCS3 mRNAs were measured by real-time PCR (upper). SOCS2 and β-actin proteins were detected by Western blot analyses (lower). (**B**–**E**) HepG2 cells were incubated with rHBeAg at different concentrations (**B**) or transfected with pCMV-HBeAg for different times (**C**). HepG2 cells were transfected with pCMV-Tag2B, pCMV-HBeAg, or pCMV-HBeAg-1896mut for 24 h (**D**) or incubated with PBS, rHBeAg, heat-inactivated HBeAg, or rHBcAg for 12 h (**E**). SOCS2 mRNAs were measured by real-time PCR (upper). SOCS2 and β-actin proteins were detected by Western blot analyses (lower). (**F**) HepG2, pBlue-SK-transfected HepG2, pHBV1.3-transfected HepG2, or HepG2.2.15 cells were harvested 24 h after transfection. SOCS2 mRNAs were measured by real-time PCR (upper). SOCS2 and β-actin proteins were detected by Western blot analyses (lower). (**G**) HepG2 cells were transfected with pBlue-SK or pHBV1.3 for different times. SOCS2 mRNA was measured by real-time PCR (upper). SOCS2 and β-actin proteins were detected by Western blot analyses (lower). (**H**) HepG2 cells were transfected with pCMV-tag2B or plasmids expressing individual HBV proteins for 48 h. SOCS2 mRNA was determined by real-time PCR (upper). SOCS2 and β-actin mRNAs were determined by semi-quantitative RT-PCR (lower). (**I**) HepG2 cells were transfected with pBlue-SK, pHBV1.3, or pHBV1.3-1896mut for 24 h. SOCS2 mRNA was determined by real-time PCR (upper). SOCS2 and β-actin proteins were detected by Western blot analyses (lower). (**J**) HepG2 cells were pretreated with each of specific kinase inhibitors for 12 h, and incubated with rHBeAg or PBS for 24 h. SOCS2 mRNAs were measured by real-time PCR. (**K**) HepG2 cells were incubated with PBS, rHBeAg, or 12-O-tetradecanoylphorbol 13-acetate (TPA) for 12 h. p-ERK, ERK, and GAPDH proteins were detected by Western blot analyses. Data shown are means ± SE; n = 3. *p < 0.05.

The mechanism by which HBeAg regulates IFN-α and IFN-λ1 was further investigated. Initially, we evaluated the roles of SOCS family members in the regulation of IFN action mediated by HBeAg in cells transfected with pCMV-HBeAg. HBeAg only enhanced the expression of SOCS2, but not SOCS1 and SOCS3, in a dose-dependent fashion (Fig. 4A). The stimulatory effect of HBeAg on SOCS2 expression was further confirmed by the following 4 results. (1) SOCS2 mRNA and protein were activated by rHBeAg in dose-dependent manners (Fig. 4B). (2) SOCS2 mRNA and protein were stimulated by HBeAg in time-dependent fashions (Fig. 4C). (3) SOCS2 mRNA and protein were enhanced by HBeAg, but not by HBeAg-1896mut (Fig. 4D). (4) SOCS2 mRNA and protein were stimulated by rHBeAg, but not by heat-inactivated rHBeAg or rHBcAg (Fig. 4E). Therefore, we revealed that HBeAg plays a specific role in activation of SOCS2.

The effect of HBV on SOCS2 expression was also determined. SOCS2 mRNA and protein were activated in pHBV1.3-transfected cells but not in pBlue-SK-transfected cells, enhanced in HepG2.2.15 cells but not in HepG2 cells (Fig. 4F), and stimulated in pHBV1.3-transfected HepG2 cells in a time-dependent manner (Fig. 4G), demonstrating that HBV stimulates SOCS2 expression in human hepatoma cells. HBV genome contains 4 overlapping open reading frames (ORFs) encoding for 7 proteins. The roles of individual viral proteins in the regulation of SOCS2 were evaluated. SOCS2 mRNA was significantly activated by HBeAg, slightly enhanced by L (PreS1/PreS2/S), but not affected by HBp, HBcAg, HBx, S, or M (PreS2/S) (Fig. 4H), indicating that HBeAg is mainly responsible for the activation of SOCS2. In addition, SOCS2 mRNA and protein were activated by HBV1.3 but not by HBV1.3-1896mut (Fig. 4I), confirming the essential role of HBeAg in regulating SOCS2.

The mechanism by which HBeAg activates SOCS2 expression was investigated using inhibitors of signaling components. SOCS2 was enhanced by rHBeAg, and this activation was not affected by DMSO, SP600125 (JNK inhibitor), LY294002 (PI3K inhibitor) or BAY-11 (NF-κB inhibitor), enhanced by H-89 (PKA inhibitor), and inhibited by PD98059 (ERK inhibitor) (Fig. 4J), suggesting that ERK signaling was involved in HBeAg-activated SOCS2 expression. Moreover, p-ERK was up-regulated by rHBeAg or TPA (Fig. 4K), indicating that HBeAg stimulates SOCS2 expression through activating ERK phosphorylation.

### SOCS2 attenuates the production of IFN receptors

**Figure 5:**
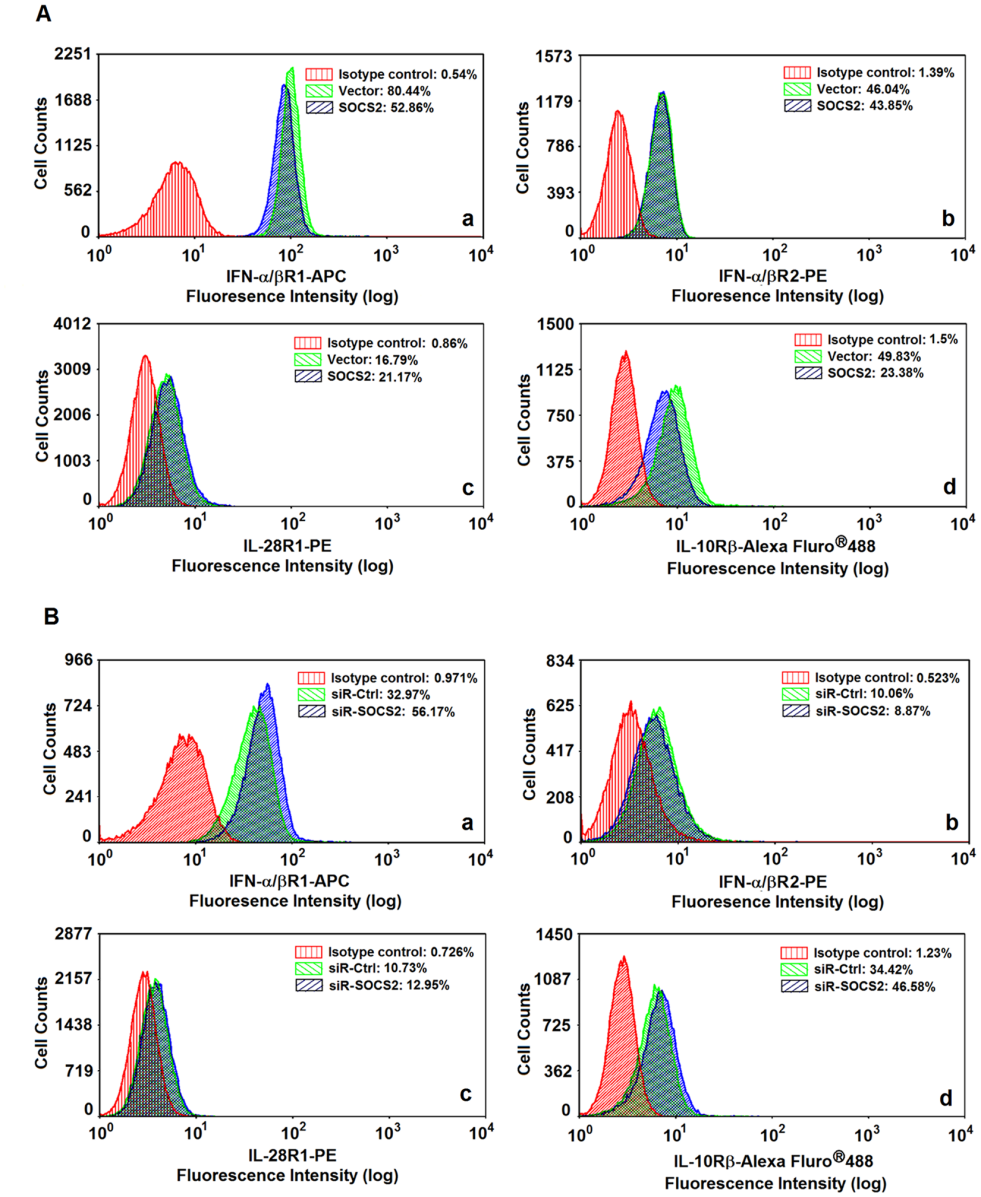
The function of SOCS2 in regulation of IFN receptors expression. (**A**) HepG2 cells were transfected with pcDNA3.1 or pcDNA3.1-SOCS2 for 48 h. Cells were Fc-blocked by treatment with human IgG prior to staining. 5 × 10^5^ to 1 × 10^6^ cells in PBS buffer were incubated with specific antibody and analyzed by flow cytometry using a FACSCalibur (Beckman Coulter) to detect the levels of IFN-α/βR1 (a), IFN-α/βR2 (b), IL-28R1 (c), and IL-10Rβ (d) proteins. (**B**) HepG2 cells were transfected with siRNA-Ctrl or siRNA-SOCS2 for 48 h. Cells were Fc-blocked by treatment with human IgG prior to staining. 5 × 10^5^ to 1 × 10^6^ cells in PBS buffer were incubated with specific antibody and analyzed by flow cytometry using a FACSCalibur (Beckman Coulter) to detect the levels of IFN-α/βR1 (a), IFN-α/βR2 (b), IL-28R1 (c), and IL-10Rβ (d) proteins.

We evaluated the effect of SOCS2 on the expression of IFN-α and IFN-λ1 receptors by overexpression or knockdown of SOCS2. HepG2 cells were transfected with pcDNA3.1 or pcDNA3.1-SOCS2. A high level of SOCS2 mRNA was detected in pcDNA3.1-SOCS2-transfected cells, indicating that transfection was efficient and SOCS2 was expressed (Fig. S4A). Flow cytometry analyses indicated that IFN-α/βR1 and IL-10Rβ were significantly reduced by SOCS2, while IFN-α/βR2 and IL-28R1 were relatively unaffected by SOCS2 (Fig. 5A). Western blot analyses confirmed that IFN-α/βR1 (IFNAR1) and IL-10Rβ were significantly reduced by SOCS2, but IFN-α/βR2 (IFNAR2) and IL-28R1 were relatively unaffected by SOCS2 (Fig. S4B). These results suggested that SOCS2 attenuates the expression of IFN-α/βR1 and IL-10Rβ. SOCS2 mRNA was obviously downregulated in siR-SOCS2 transfected cells, indicating that siR-SOCS2 was effective (Fig. S4C). IFN-α/βR1 and IL-10Rβ were up-regulated by siR-SOCS2 (Fig. 5Ba and Bd), while IFN-α/βR2 and IL-28R1 were relatively unaffected by siR-SOCS2 (Fig. 5Bb and Bc), suggesting that knockdown of SOCS2 upregulates IFN-α/βR1 and IL-10Rβ. Taken together, we revealed that SOCS2 represses IFN-α and IFN-λ1 action by inhibiting their receptors, IFN-α/βR1 and IL-10Rβ.

### SOCS2 reduces TYK2 stability and phosphorylation

**Figure 6:**
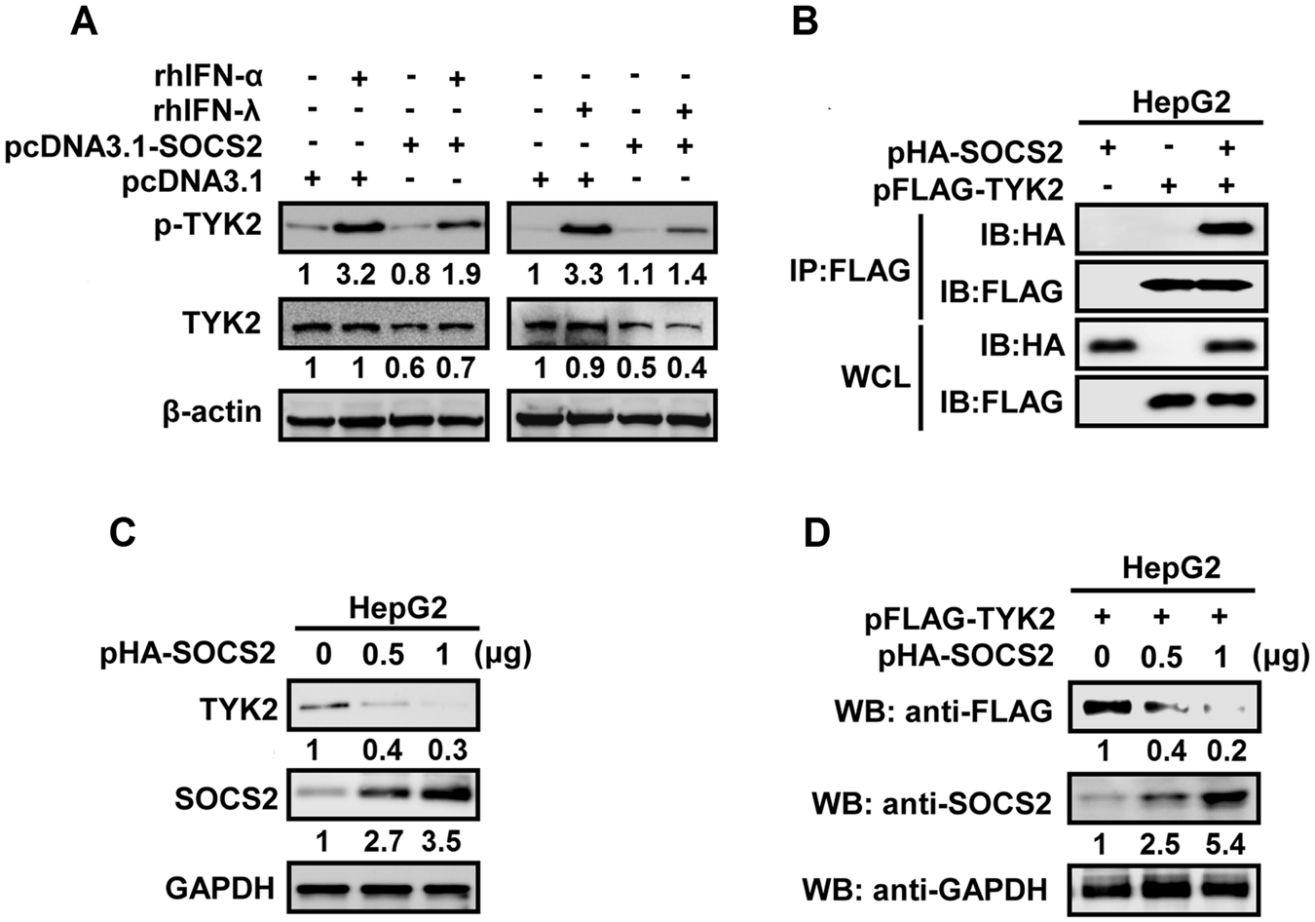
The effect of SOCS2 on IFN-induced TYK2 phosphorylation and protein stability. (**A**) HepG2 cells were transfected with pcDNA3.1 or pcDNA3.1-SOCS2 for 48 h and treated with rhIFN-α at 300 U/ml or rhIFN-λ1 at 100 ng/ml for 30 min. Cells were harvested and lysed. p-TYK2, TYK2, and β-actin proteins were detected by Western blot analyses. (**B**) HepG2 cells were transfected with pHA-SOCS2 or pFLAG-TYK2 or co-transfected with pHA-SOCS2 and pFLAG-TYK2 for 48 h. Cells were harvested and lysed, and then incubated with protein A-Sepharose CL-4B and appropriate antibodies. Immunoprecipitated proteins, HA-SOCS2 and FLAG-TYK2, were separated by SDS-PAGE, transferred onto PVDF membranes (Millipore), and detected by Western blot analyses. (**C**) HepG2 cells were transfected with pHA-SOCS2 at indicated concentrations for 48 h. Cells were harvested and lysed, and TYK2, SOCS2, and GAPDH proteins were detected by Western blot analyses. (**D**) HepG2 cells were co-transfected with pFLAG-TYK2 and pHA-SOCS2 at indicated concentrations for 48 h. Cells were harvested and lysed, and FLAG-TYK2, SOCS2, and GAPDH proteins were detected by Western blot analyses.

Since IFN-α/βR1 and IL-10Rβ are associated with TYK2, we determined whether the reduction of IFN-α/βR1 and IL-10Rβ mediated by SOCS2 leads to the dysregulation of TYK2. Cells were transfected with pcDNA3.1 or pcDNA3.1-SOCS2 and treated with rhIFN-α or rhIFN-λ1. p-TYK2 was enhanced by rhIFN-α and rhIFN-λ1 but reduced by SOCS2, and total TYK2 was also down-regulated by SOCS2 (Fig. 6A), suggesting that SOCS2 attenuates TYK2 production and activation.

Since SOCS1 inhibits type I IFN by binding to IFN receptor-associated TYK2^34^, it is reasonable for us to speculate that SOCS2 may interact with TYK2. SOCS2 was co-immunoprecipitated with TYK2 in the cells co-transfected with pHA-SOCS2 and pFLAG-TYK2 (Fig. 6B), confirming that SOCS2 interacts with TYK2. We then investigated the role of SOCS2 in the regulation of endogenous TYK2 and demonstrated that the level of TYK2 protein was gradually decreased by SOCS2 in a dose-dependent manner (Fig. 6C). We further evaluated the effect of SOCS2 on TYK2 protein stability in HepG2 cells and confirmed that FLAG-TYK2 protein was gradually degraded as the concentration of SOCS2 increased (Fig. 6D). Taken together, we demonstrated that SOCS2 interacts with TYK2 to attenuate its stability.

### Knockdown of SOCS2 rescues HBeAg-mediated repression of STAT1

**Figure 7:**
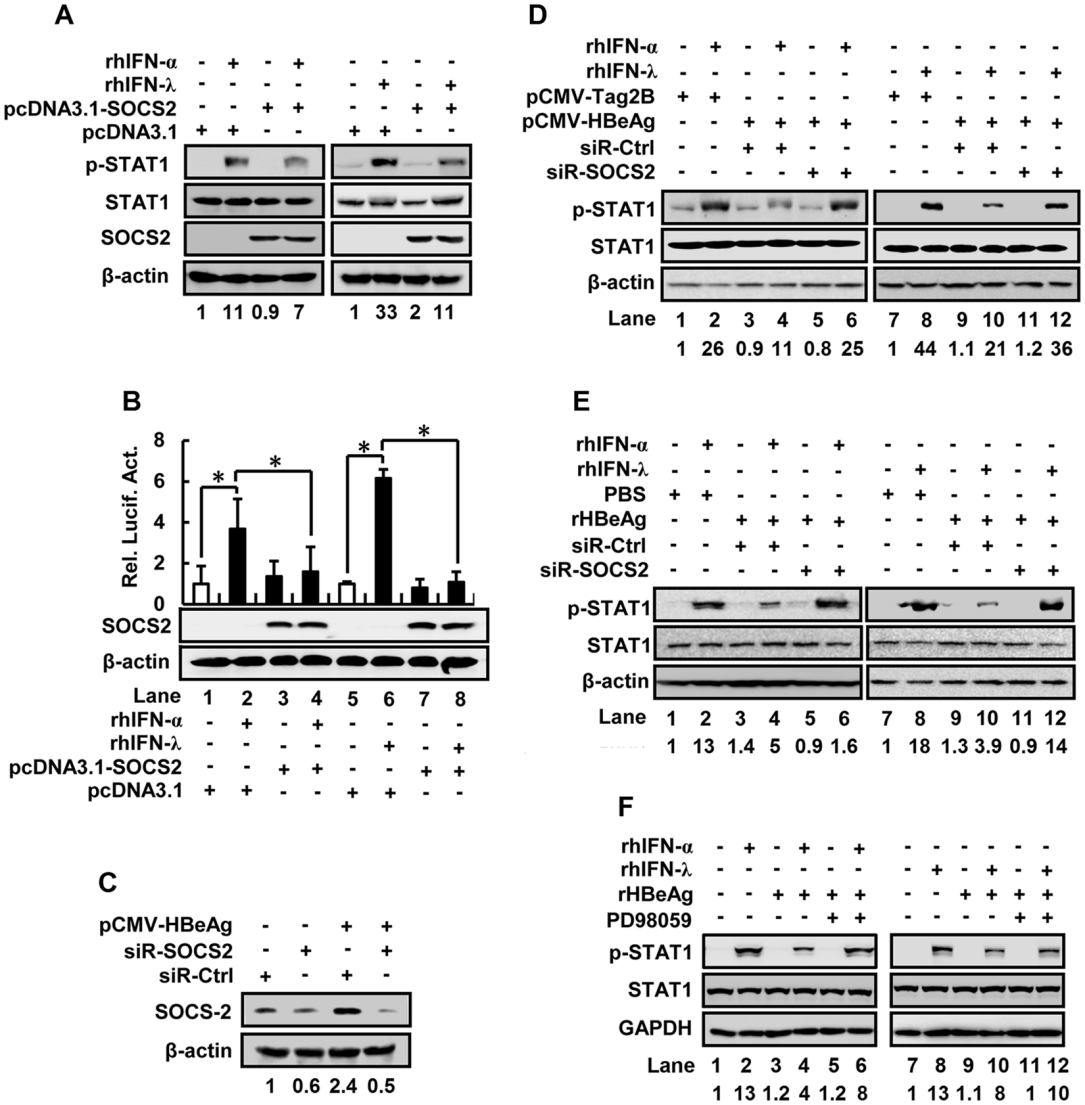
The role of SOCS2 in the repression of IFN signaling mediated by HBeAg. (**A**) HepG2 cells were transfected with pcDNA3.1 or pcDNA3.1-SOCS2 for 48 h and treated with rhIFN-α at 300 U/ml or rhIFN-λ1 at 100 ng/ml for 30 min. Cells were harvested and lysed, and p-STAT1, STAT1, SOCS2, and β-actin proteins were detected by Western blot analyses. (**B**) HepG2 cells were co-transfected with pISRE-Luc and pcDNA3.1 or pcDNA3.1-SOCS2 for 24 h and treated with rhIFN-α or rhIFN-λ1 for another 12 h. The activity of IFN-stimulated response element (ISRE) was measured by luciferase activity assays (upper panel). Data shown were means ± SE; n = 3. *p < 0.05. To confirm the expression of SOCS2 in pcDNA3.1-SOCS2-transfected cells, SOCS2 and β-actin proteins were detected by Western blot analyses (lower panel). (**C**) Determination of the efficiency of siRNA-SOCS2 in HepG2 cells. HepG2 cells were co-transfected with pCMV-tag2B or pCMV-HBeAg and the siRNA specific to SOCS2 (siR-SOCS2) or its control siRNA (siR-Ctrl) for 48 h. Cells were harvested and lysed, and SOCS2 and β-actin proteins in the cell lysates were detected by Western blot analyses. (**D**) HepG2 cells were co-transfected with pCMV-Tag2B or pCMV-HBeAg and siR-SOCS2 or siR-Ctrl for 48 h, and treated with rhIFN-α or rhIFN-λ1 for 30 min. (**E**) HepG2 cells were transfected with siR-Ctrl or siR-SOCS2 for 24 h, and incubated with rHBeAg (50 ng/ml) or PBS for another 24 h, and then treated with rhIFN-α or rhIFN-λ1for 30 min before harvest. (**F**) HepG2 cells were pretreated with or without PD98059 for 12 h, and incubated with rHBeAg (50 ng/ml) or PBS for 24 h, and then treated with rhIFN-α or rhIFN-λ1 for 30 min before harvest. (**D**–**F**) Cells were harvested and lysed, and p-STAT1, STAT1, β-actin, and GAPDH proteins were detected by Western blot analyses.

The effect of SOCS2 on IFN-induced activation of STAT1 was further evaluated. The results showed that p-STAT1 was up-regulated by rhIFN-α or rhIFN-λ1, but down-regulated by SOCS2 (Fig. 7A). To determine the role of SOCS2 in the regulation of ISRE-dependent genes, cells were co-transfected with pcDNA3.1-SOCS2 and pISRE-Luc, in which the expression of luciferase (Luc) gene is under the control of ISRE, and treated with rhIFN-α or rhIFN-λ1. ISRE activity was stimulated by rhIFN-α and rhIFN-λ1, but repressed by SOCS2 (Fig. 7B). Therefore, SOCS2 represses the actions of IFN-α and IFN-λ1 through attenuating STAT1 phosphorylation and ISRE activity.

The effect of SOCS2 on HBeAg-mediated regulation of IFN/JAK/STAT signaling was also evaluated by knockdown of SOCS2. SOCS2 was downregulated by siR-SOCS2, but not by siR-Ctrl (Fig. 7C), indicating that siR-SOCS2 was effective and specific. p-STAT1 was enhanced by rhIFN-α or rhIFN-λ1, but IFN-induced p-STAT1 was repressed by HBeAg (Fig. 7D). Interestingly, HBeAg-mediated repression of p-STAT1 was rescued by siR-SOCS2 (Fig. 7D). Consistently, p-STAT1 was enhanced by rhIFN-α or rhIFN-λ1, but IFN-induced p-STAT1 was repressed by rHBeAg (Fig. 7E). Similarly, rHBeAg-mediated repression of p-STAT1 was recovered by siR-SOCS2 (Fig. 7E). Taken together, we demonstrated that knockdown of SOCS2 results in the recovery of HBeAg-mediated repression of STAT1 phosphorylation.

Since HBeAg activates SOCS2 through ERK signaling, and PD98059 (an ERK inhibitor) represses HBeAg-mediated activation of SOCS2 (Fig. 4J), we further determined whether the effect of HBeAg on repression of IFN signaling was due to the activation of SOCS2. Cells were pretreated with PD98059, incubated with rHBeAg, and then treated with IFN-α or IFN-λ1. p-STAT1 was enhanced by rhIFN-α or rhIFN-λ1, but IFN-induced p-STAT1 was repressed by rHBeAg (Fig. 7F). Moreover, rHBeAg-mediated downregulation of p-STAT1 was attenuated by PD98059 (Fig. 7F), indicating that inhibition of SOCS2 can rescue HBeAg-mediated repression of IFN signaling. Taken together, we provided strong evidence to support that SOCS2 plays an important role in HBeAg-mediated repression of IFN-α and IFN-λ1 actions.

## Discussion

HBV does not induce a substantial IFN-α/β response in the liver^34^, however, its replication is sensitive to IFN-α/β and IFN-γ produced by NK, NKT, and T cells^35^. IFN-α is used therapeutically to treat HBV infection but has a poor response rate. Thus, HBV must develop strategies to counteract IFN actions and ensure persistent infection. Here, initially, we showed that HBV impairs IFN activity by hijacking the IFN/JAK/STAT pathway through HBeAg. It is known that HBeAg is not required for HBV replication and its exact function is unclear, but may play a role in chronic HBV infection^17, 18^. The emergence of HBeAg-negative variants correlates with an exacerbation of liver injury in some patients^20, 21^. HBeAg modulates host immune response during CHB progression^22^, suppresses TLR-induced IFN-β and ISG production in liver cells^23^, and inhibits IL-18 signaling and IFN-γ expression in NK and hepatoma cells^36^. In response to HBV infection-established persistent infections, virus-specific CD4 and CD8 T cells are physically deleted or persist in an attenuated (termed exhausted) developmental program unable to proliferate to viral antigens or produce important antiviral and immunostimulatory cytokines (including IFNγ, TNFα, and IL-2)^37^. HBeAg appears to be critical in determining the outcome of immunotherapies in chronic HBV patients. A pDC-based immunotherapeutic approach could be of interest in attempts to restore functional antiviral immunity, which is critical for the control of the virus in chronic HBV patients^38^. However, despite the important clinical and cellular implications, the molecular mechanism by which HBeAg regulates host immunity remains largely unknown. A previous study provided a molecular mechanism describing HBeAg immunomodulation of innate immune signal transduction pathways *via* interaction and targeting of TLR-mediated signaling pathways^39^. Here, our results demonstrated that HBeAg represses IFN action and IFN/JAK/STAT signaling.

Subsequently, we revealed that SOCS2 is required for the function of HBeAg in the repression of IFN activity. Members of the SOCS family are negative regulators of cytokine signaling pathway^26–28^. SOCS1 inhibits IFN-γ signaling *via* direct interaction with IFNGR1 or JAK kinases^40^, and represses type I IFN signaling via the interferon alpha receptor (IFNAR1)-associated TYK2^41^. SOCS3 attenuates cell signaling *via* binding directly to the cytokine receptor subunit gp130^42^. HBV regulates the expression SOCS1 and SOCS3 in mouse liver^43^. We noticed that our result revealing HBeAg activates SOCS2 expression in human hepatoma cells is contrary to the previous report showing HBV had not effect on SOSC-2 expression in mouse liver^43^. We speculated that the discrepancy between these results may be due to the differences in cell phenotype, cellular responses, surface receptors, and cellular functions between human hematoma cells and mouse liver cells.

Moreover, we demonstrated that SOCS2 subsequently interacts with TYK2 to reduce the protein stability. In addition to the facilitation of IFN signaling by enhancing the interaction between STAT1 and IFN receptor, TYK2 is also required for maintaining IFN receptors on the cell membrane. Thus, it is reasonable for us to speculate that interaction of SOCS2 with TYK2 may result in the dissociation of TYK2 from IFN receptor, leading to a reduction in the receptors. Interestingly, we confirmed that SOCS2 indeed reduces IFN-α/βR1 production, suppresses TYK2 phosphorylation, and attenuates STAT1 nuclear translocation, which lead to the repression of ISGs production.

The effectiveness of IFN-α treatment between HBeAg-positive and HBeAg-negative patients remains uncertain. Most studies have shown that approximately 30–40% of HBeAg-positive patients respond to IFN-α therapy. However, the rate to IFN-α therapy for HBeAg-negative patients is divergent: some studies reported that IFN-α response rate is up to 50%^44–46^, whereas others showed long-term IFN-α response rate is less than 10%^47–50^. Thus, we could not neglect the roles of other viral proteins, including HBsAg and HBp^23, 51, 52^, in the regulation of IFN-α actions. In contrast to type I IFNs, IFN-λ is elevated in PBMCs of patients with CHB, and HBV is sensitive to IFN-λ in cell culture models^8, 53–55^. The weaker and prolonger antiviral responses induced by IFN-λ may have implications for the therapeutic use of IFN-λ^56, 57^. Low transaminase levels, high viral replication, long duration of disease, and low inflammatory score in liver histology have been reported to be associated with low response rates to IFN therapy^58–61^. We demonstrated that SOCS2 also inhibits the expression of IFN-λ receptor and IFN-λ activity, providing a better understanding how HBV resists IFN-α and IFN-λ treatment.

**Figure 8:**
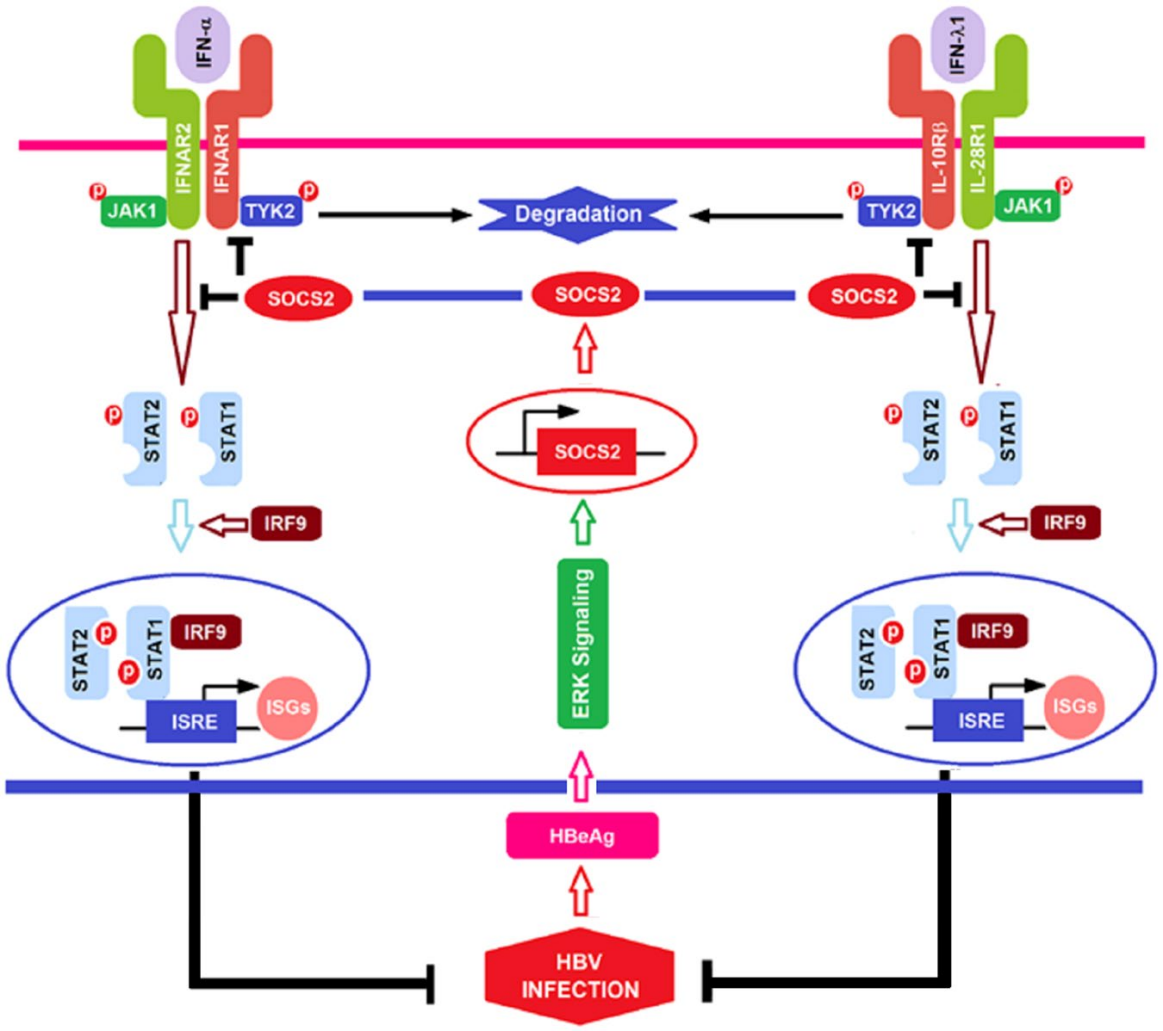
A proposed mechanism by which HBV hijacks IFN/JAK/STAT pathway *via* activating SOCS2 to facilitate immune evasion and viral infection. During hepatitis B virus (HBV) infection, the extracellular viral protein (hepatitis B e antigen, HBeAg) activates the cellular factor (suppressor of cytokine signaling 2, SOCS2) expression through regulating the extracellular regulated protein kinase (ERK) signaling. Activated SOCS2 subsequently hijacks the IFN/JAK/STAT pathway to reduce tyrosine kinase 2 (TYK2) stability and phosphorylation, downregulate interferon-α/β receptor 1 (IFN-α/βR1, IFNAR1) and interferon-λ1 receptor (cytokine receptor family 2 member 4, CRF2-4 or IL-10Rβ) production, attenuate signal transducer and activator of transcription 1 (STAT1) phosphorylation and nucleus translocation, and finally block IFN-stimulated genes (ISGs) expression, which results in the facilitation of HBV immune evasion, persistent infection, and possible pathogenesis.

In conclusion, we reveal a novel mechanism by which HBeAg and SOCS2 are coordinated to enhance HBV infection by hijacking the IFN/JAK/STAT pathway and attenuating IFN action (Fig. 8). HBeAg initially activates SOCS2 that subsequently hijacks the IFN/JAK/STAT signaling to reduce TYK2 stability and phosphorylation, down-regulate IFN receptors production, attenuate STAT1 phosphorylation and nucleus translocation, and finally block ISGs production, which results in the facilitation of HBV immune evasion and persistent infection.

## Methods

### Reagents

Recombinant human IFN-λ1 (rhIFN-λ1) was purchased from eBioscience (San Diego, CA). Recombinant human IFN-α (rhIFN-α) was purchased from SanSheng Biotech (Shenyang, China). Recombinant hepatitis B e antigen (rHBeAg) was obtained from ViroStat (Portland, ME). Recombinant HBV core protein (rHBcAg) was purchased from ProSpec (East Brunswick, NJ). Recombinant human interleukin 6 (rhIL-6), recombinant human interleukin 4 (rhIL-4) and recombinant human IFN-γ (rhIFN-γ) were purchased from Peprotech, Inc. (Rocky Hill, NJ). 12-O-tetradecanoylphorbol 13-acetate (TPA) was purchased from Sigma-Aldrich (St. Louis, MO). Antibody specific to HBeAg was purchased from Abcam (Cambridge, MA). Antibodies specific to β-actin, STAT1, phospho-STAT1, JAK1, phospho-JAK1, TYK2, phospho-TYK2, phospho-ERK, ERK, IL-28R1, IL-10Rβ, IFN-α/βRα and IFN-α/βRβ were purchased from Santa Cruz Biotechnology (Santa Cruz, CA). Antibodies specific to SOCS2, phospho-STAT3, STAT3, phospho-STAT6, and STAT6 were purchased from Cell Signaling Technology (Beverly, MA). Antibodies to Lamin A were obtained from Epitomics Company (Burlingame, CA). Flow cytometry antibodies specific to IFN-α/βR1, IFN-α/βR2, IL-28R1, and IL-10Rβ were purchased from R&D Systems. PD98059 (ERK inhibitor), H-89 (PKA inhibitor), LY294002 (PI3K inhibitor), SP600125 (JNK inhibitor), and BAY-11 (NF-κB inhibitor) were purchased from Sigma-Aldrich (St. Louis, MO). All inhibitors were dissolved in dimethyl sulfoxide (DMSO).

### Cell culture

Human hepatoma cells HepG2, Huh7, HepaAD38 and HepG2-NTCP were grown in DMEM supplemented with 10% heat-inactivated fetal calf serum, 100 U/ml penicillin, and 100 mg/ml streptomycin sulfate at 37 °C with 5% carbon dioxide. HepG2.2.15 cells (derived from HepG2 cells) carrying the HBV genome (ayw) were maintained in DMEM containing 400 μg/ml G418 and supplemented with 10% heat-inactivated FCS, 100 U/ml penicillin, and 100 mg/ml streptomycin sulfate at 37 °C with 5% carbon dioxide.

### Viruses and infection

For infection of HepG2-NTCP cells (provided by Ying Zhu, Wuhan University, China), HBV inoculums were concentrated 100-fold from the supernatants of HepaAD38 cells (provided by Ying Zhu of Wuhan University, China) by ultracentrifugation. For infection, HepG2-NTCP cells were seeded onto collagen I-coated plates in Dulbecco’s modified Eagle medium (DMEM) for 6 h, and the medium was then changed to PMM with 2% fetal bovine serum (FBS). PMM is Williams’ E medium supplemented with ITS (catalog no. I3146; Sigma), 2 mM L-glutamine, 10 ng/ml of human epidermal growth factor (EGF), 18 μg/ml of hydrocortisone, 40 ng/ml of dexamethasone, 2% dimethyl sulfoxide (DMSO), 100 U/ml of penicillin, and 100 μg/ml of streptomycin for 12 h. Cells were then infected with 1,000 GEq per cell of HBV in PMM containing 4% (wt/vol) polyethylene glycol 8000 (PEG 8000) for 16 h. The virus-containing medium was removed, and cells were washed four times and further incubated in PMM. The medium was changed every other day^62, 63^.

### Plasmid construction

A DNA fragment containing a 1.3-fold length of HBV genome (ayw subtype) was amplified from HepG2.2.15 cells and inserted into pBluescript II (Invitrogen, San Diego) to generate pHBV1.3 as previously described^64^. pHBV1.3-1896mut carrying a G to A mutation at nucleotide 1896 (resulting in a stop codon) in HBV genome, leading to abrogation of HBeAg synthesis but not affecting HBV replication, was constructed as described previously^65^. Individual HBV genes were amplified from pHBV1.3 and sub-cloned into pCMV-Tag2B (Invitrogen) to generate pCMV-L, pCMV-M, pCMV-S, pCMV-HBeAg, pCMV-HBcAg, pCMV-HBx, and pCMV-HBp as described previously^66^. The coding sequence of the mutant HBV envelope protein was amplified from pHBV1.3-1896mut by PCR using the primers HBeAg-1896mut sense (5′-TTGGTGGAATTCCTGCAGCCCGGGGGA-3′) and HBeAg-1896mut antisense (5′-TTTACTCGAGGGGGGGCCCGGTACCTT-3′). The PCR product was then inserted into pCMV-Tag-2B to create pCMV-HBeAg-1896mut. The plasmid pCMV-HBeAg-1896mut cannot express HBeAg or HBcAg. The TYK2 gene was amplified from cDNA of Hep3B cells using the primers TYK2 sense 5′-TTAGAATTCATGCCTCTGCGCCACTGG-3′ and TYK2 antisense 5′-CCGAAGCTTTCAGCACACGCTGAACACTG-3′, and the PCR product was sub-cloned into pCMV-Tag2B to generate pFLAG-TYK2. The SOCS2 gene is amplified from cDNA of HEK293 cells using the primers SOCS2 sense 5′-GACTGCTAGCCATGACCCTGCGGTGCCTTGAG-3′ and SOCS2 antisense 5′-AGTCCTCGAGTTATACCTGGAATTTATATTCTTCCAAG-3′, and the PCR product was sub-cloned into pcDNA3.1 (Invitrogen) to generate pcDNA3.1-SOCS2. The SOCS2 gene was amplified using the primers HA-SOCS2 sense 5′-TCGGATCCATGACCCTGCGGTGCCTTGAGCCCT-3′ and HA-SOCS2 antisense 5′-CAGTCGACTACCTGGAATTTATATTCTTCCAAG-3′, and the PCR product was sub-cloned into pRK3HA to generate pHA-SOCS2. Small interfering RNA (siRNA) specific to SOCS2 (siR-SOCS2) and its negative control (siR-NC) were purchased from RiBo Biotech (GuangzhouRiBo Biotech). The IFN stimulation response element (ISRE)-luciferase reporter plasmid (pISRE-Luc) was a gift from Dr. Hongbing Shu of Wuhan University, China.

### Luciferase assay

HepG2 cells were co-transfected with reporter plasmids and their corresponding expression plasmids. Cells were lysed with luciferase cell culture lyses reagent (Promega, Madison, WI). Cell lysates and luciferase assay substrate (Promega) were mixed, and the light intensity was detected by a luminometer (Turner T20/20). Assays were performed in triplicate and expressed as means relative to the vector control (100%).

### Semi-quantitative RT-PCR analysis

Total RNA was isolated from the cells using TRIzol reagent (Invitrogen, Carlsbad, CA), treated with DNaseI, and reverse-transcribed with MLV reverse transcriptase (Promega) using random primers (Takara). PCR is performed in 25 μl reactions with the following detection primer pairs: SOCS2 sense, 5′-TCGTTTTGGGGTACCCTGTGAC-3′ and SOCS2 antisense, 5′-GAAAGTTCCTTCTGGCGCCTCT-3′. β-actin is amplified by PCR for normalization in all experiments.

### Real-time PCR

Total RNA was extracted from the cells with TRIzol reagent following the manufacturer’s instructions (Invitrogen). Real-time PCR analysis was performed using the Roche LC480 and SYBR RT-PCR kits (DBI Bioscience) in a reaction mixture of 20 μl containing 0.5 mM of each PCR primer, 10 μl of SYBR Green PCR master mix, 1 μl of DNA diluted template, and RNase-free water to complete the 20 μl volume. Real-time primers are as follows: SOCS1 sense, 5′-CACCTTCTTGGTGCGCG-3′; SOCS1 antisense, 5′-AAGCCATCTTCACGCTGAGC-3′; SOCS2 sense, 5′-GGATGGTACTGGGGAAGTATGACTG-3′; SOCS2 antisense, 5′-AGTCGATCAGATGAACCACACTGTC-3′; SOCS3 sense, 5′-GCTCCAAAAGCGAGTACCAGC-3′; SOCS3 antisense, 5′-AGTAGAATCCGCTCTCCTGCAG-3′. Primers for 2′5′OAS, PKR, and GAPDH were previously described^67^. Data were normalized to the level of GAPDH expression in each sample, as described earlier.

### HBV protein assays

At 48 h post-transfection, the HBeAg and hepatitis B s antigen (HBsAg) protein levels in cell culture medium were determined by ELISA using an HBV HBeAg or HBsAg diagnostic kit (Shanghai KeHua Biotech).

### Analysis of HBV DNA

HBV capsid-associated DNA was extracted from the cells as described previously, with modifications^64^. Equivalent amounts of HepG2 cells were homogenized in 1 ml lyses buffer (50 mM Tris, pH7.5, 0.5% Nonidet P-40, 1 mM EDTA, and 100 mM NaCl) and mixed gently at 4 °C for 1 h. Next, 10 μl of 1 M MgCl_2_ and 10 μl of 10 mg/ml DNase were added and incubated for 2 h at 37 °C. Viral cores were precipitated by adding 35 μl of 0.5 M EDTA and 225 μl of 35% polyethylene glycol and incubated at 4 °C for 30 min. They were then concentrated by centrifugation, and the pellets were resuspended in 10 mM Tris, 100 mM NaCl, 1 mM EDTA, 1% SDS, and 20 μl proteinase K (25 mg/ml) and incubated overnight. Viral DNA released from the lysed cores was extracted with phenol and chloroform, precipitated with isopropanol, and resuspended in Tris-EDTA. Resuspended HBV capsid-associated DNA was quantified by real-time PCR as described by the manufacturer (PG Biotech, Shenzhen, China). Primers used in real-time PCR were as follows: P1, 5′-ATCCTGCTGCTATGCCTCATCTT-3′ and P2, 5′-ACAGTGGGGAAAGCCCTACGAA-3′. The probe was 5′-TGGCTAGTTTACTAGTGCCATTTTG-3′. PCR was carried out and analyzed using a Roche LC480 instrument.

### Nuclear extraction

Cells were incubated in serum-free media for 24 h, washed twice with PBS, and scraped into 1 ml cold PBS. Cells were harvested by centrifugation for 15 s and incubated in two packed cell volumes of buffer A (10 mM HEPES, pH8.0, 0.5% Nonidet P-40, 1.5 mM MgCl_2_, 10 mM KCl, 0.5 mM DTT, and 200 mM sucrose) for 5 min at 4 °C with flipping of the tube. The crude nuclei were collected by centrifugation for 30 s, and the pellets were rinsed with buffer A, resuspended in buffer B (20 mM HEPES, pH7.9, 1.5 mM MgCl_2_, 420 mM NaCl, 0.2 mM EDTA, and 1.0 mM DTT), and incubated on a shaking platform for 30 min at 4 °C. Nuclei were centrifuged for 5 min, and the supernatants were diluted 1:1 with buffer C (20 mM HEPES, pH7.9, 100 mM KCl, 0.2 mM EDTA, 20% glycerol, and 1 mM DTT). Cocktail protease inhibitor tablets were added to each type of buffer. Nuclear extracts were snap-frozen in liquid nitrogen and stored at −70 °C until use.

### Western blot analysis

Whole-cell lysates were prepared by lysing cells with PBS containing 0.01% Triton X-100, 0.01% EDTA, and a 10% protease inhibitor mixture (Roche). The protein concentration was determined by Bradford assay (Bio-Rad). The cell lysates (100 mg) were electrophoresed in a 12% SDS-PAGE gel and transferred to a nitrocellulose membrane (Amersham). Nonspecific sites were blocked with 5% nonfat dried milk before being incubated with an antibody used in this study. Protein bands were detected by using SuperSignal Chemiluminescent substrate (Pierce, Rockford, IL).

### Immunoprecipitation

At 24 h post-transfection, cells were harvested and lysed. Co-IP was carried out using appropriate tag antibody and protein A Sepharose (GE Healthcare). After several times of washes, precipitated protein were eluted in SDS loading buffer and separated by SDS-PAGE, transferred onto PVDF membranes (Millipore) and detected in Western blots with appropriate antibodies.

### Flow cytometry

HepG2 cells were Fc-blocked by treatment with 1 mg human IgG per 10^5^ cells for 15 min at 4 °C prior to staining. In total, 5 × 10^5^ to 1 × 10^6^ cells in PBS buffer supplemented with 2% BSA are incubated with 1 mg/ml specific antibody for 1 h at 4 °C. Cells were washed twice with PBS buffer supplemented with 2% BSA, and 2.5 × 10^4^ cells/sample were then analyzed by flow cytometry using a FACS Calibur (Beckman Coulter).

### Statistical analysis

All experiments were reproducible and carried out in duplicate or quadruplicate. Each set of experiments was repeated at least three times with similar results, and representative experiments were shown. The results were presented as means. Student’s t-test for paired samples was used to determine statistical significance. Differences were considered statistically significant at a p value of ≤0.05.

## Electronic supplementary material

Supplementary Figures
