# Supplementary material for: Hepatitis B Virus e Antigen Activates the Suppressor of Cytokine Signaling 2 to Repress Interferon Action
Source: Sci Rep. 2017 May 11;7:1729. doi: 10.1038/s41598-017-01773-6 (PMC5431827; doi:10.1038/s41598-017-01773-6)
Supplement: Supplementary file 1 — Supplementary Figures [file 41598_2017_1773_MOESM1_ESM.doc]

**Hepatitis B Virus e Antigen Activates the Suppressor of Cytokine Signaling 2 to Repress Interferon Action**

Yi Yu^1,2^, Pin Wan^1^, Yanhua Cao^1^, Wei Zhang^1^, Junbo Chen^1^, Li Tan^1^, Yan Wang^1^, Zhichen Sun^1^, Qi Zhang^1^, Yushun Wan^1^, Ying Zhu^1^, Fang Liu^1^, Kailang Wu^1^,**, Yingle Liu^1^,**, and Jianguo Wu^1^,*

^1^State Key Laboratory of Virology and College of Life Sciences, Wuhan University, Wuhan 430071, China; ^2^Life Sciences Institute, Zhejiang University, Hangzhou, China

**Supplementary Information**

**Supplementary Figures and Legends**


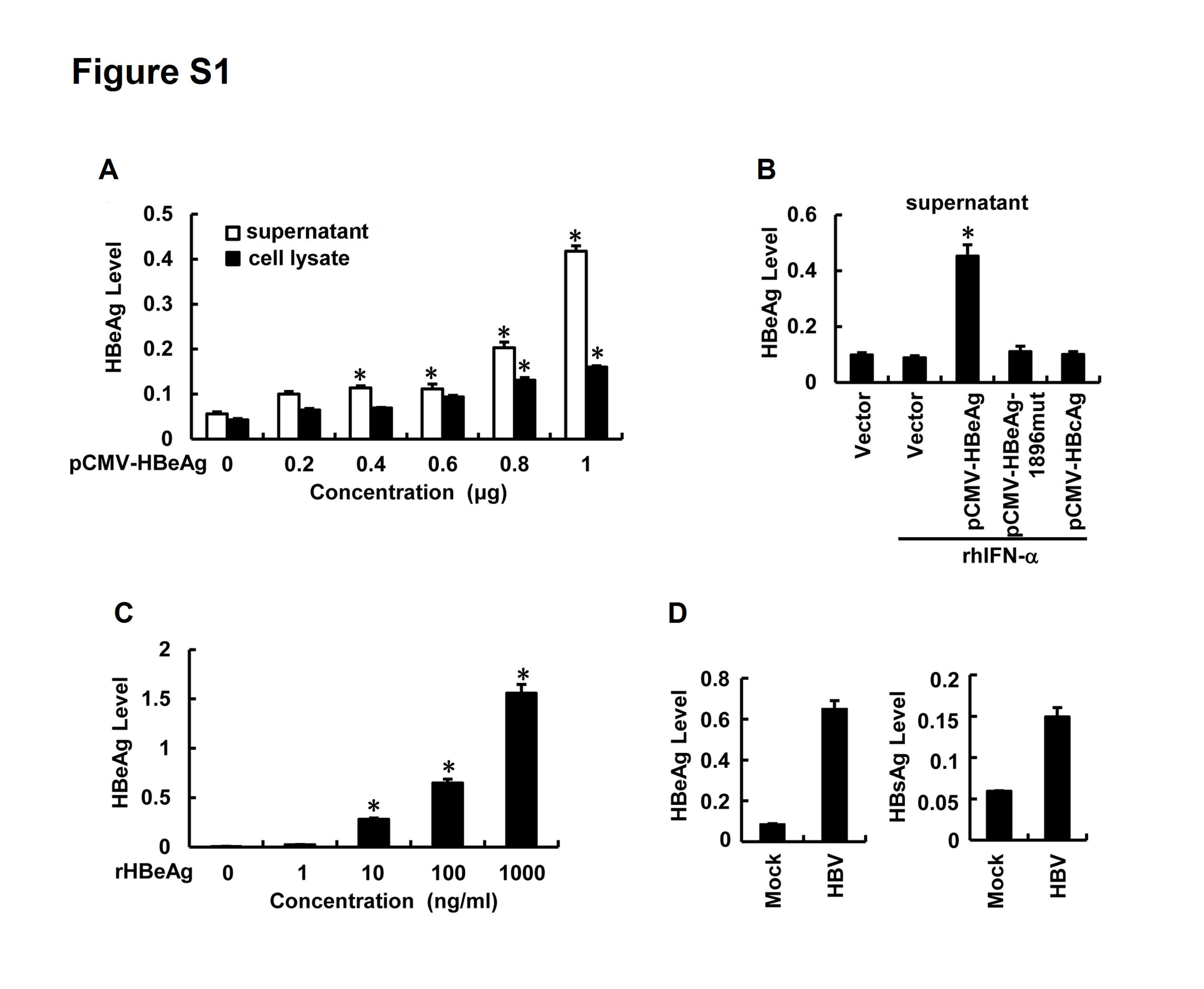


**Supplementary Figure S1. The effect of HBeAg in the phosphorylation and nuclear translocation of STAT1 induced by IFN-α and IFN-λ1.**

(**A**) HepG2 cells were transfected with different concentrations of pCMV-HBeAg for 48 h. The levels of HBeAg in cell culture supernatant and cell lysates were determined by ELISA. (**B**) HepG2 cells were transfected with pCMV-Tag2B, pCMV-HBeAg, pCMV-HBeAg-1896mut, or pCMV-HBcAg for 48 h and then treated with rhIFN-α at 300 U/ml for 30 min. The levels of HBeAg protein in cell culture supernatant were determined by ELISA. (**C**) HepG2 cells were incubated with different concentrations of rHBeAg for 24 h. The levels of HBeAg in cell culture supernatant were determined by ELISA. Data shown were means ± SE; n = 3. *p < 0.05. (**D**) HepG2-NTCP cells were mock infected or infected with HBV at 1,000 GEq per cell for 3 days. The levels of HBeAg and HBsAg in cell culture medium were determined by ELISA.


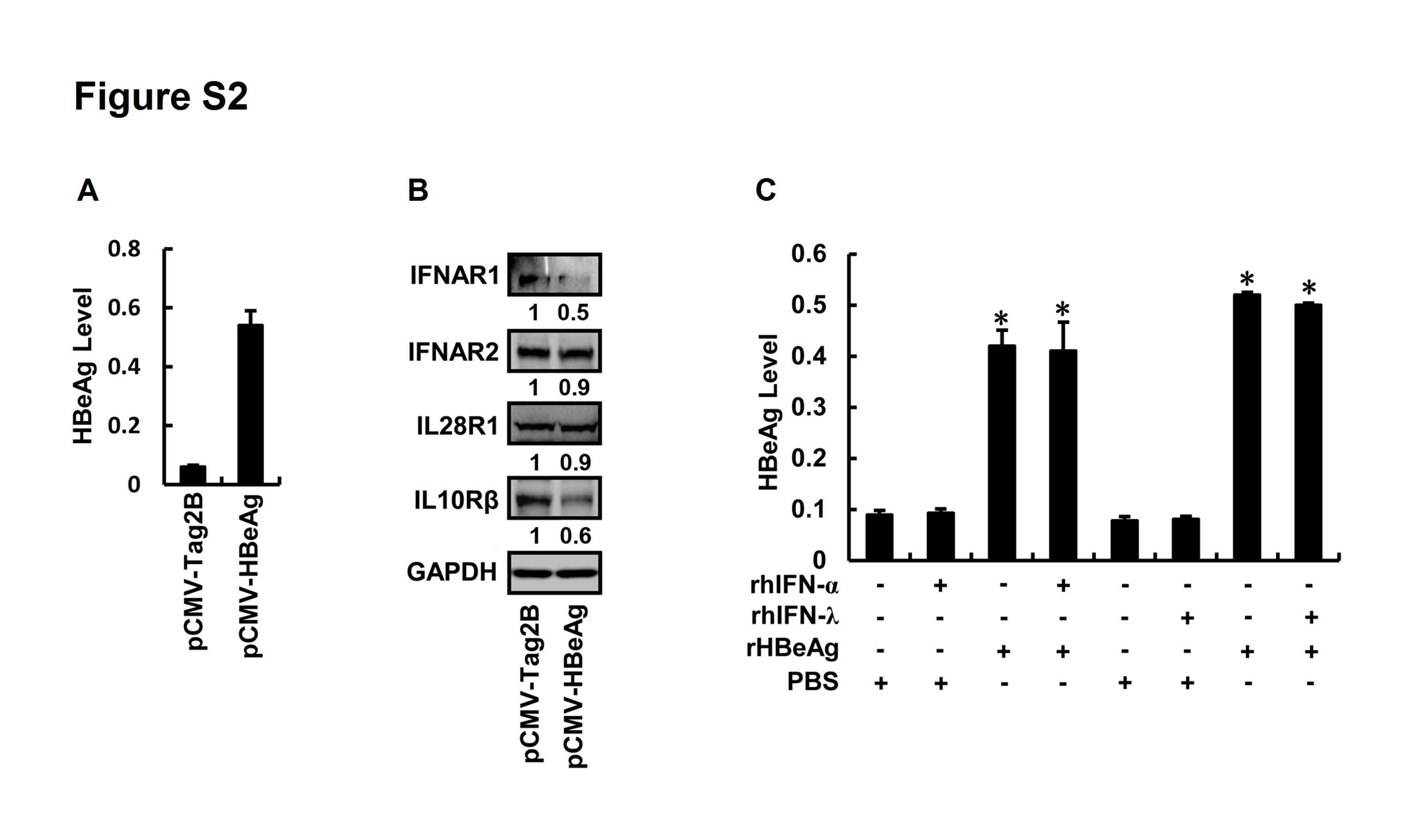


**Supplementary Figure S2. The role of HBeAg in the regulation of IFN receptors expression, TYK2 stability and TYK2 phosphorylation induced by IFN-α and IFN-λ1.**

(**A**) HepG2 cells were transfected with pCMV-Tag2B or pCMV-HBeAg for 48 h. The levels of HBeAg in cell culture medium were determined by ELISA. (**B**) HepG2 cells were transfected with pCMV-Tag2B or pCMV-HBeAg for 48 h. Cells were harvested and lysed, and IFNAR1, IFNAR2, IL-28R1 and IL-10Rβ proteins in the cell lysates were detected by Western blot analyses. (**C**) HepG2 cells were incubated with PBS or rHBeAg at 50 ng/ml for 24 h, and then treated with rhIFN-α at 300 U/ml or rhIFN-λ1 at 100 ng/ml for 30 min. The levels of HBeAg in cell culture supernatant were determined by ELISA. Data shown were means ± SE; n = 3. *p < 0.05.


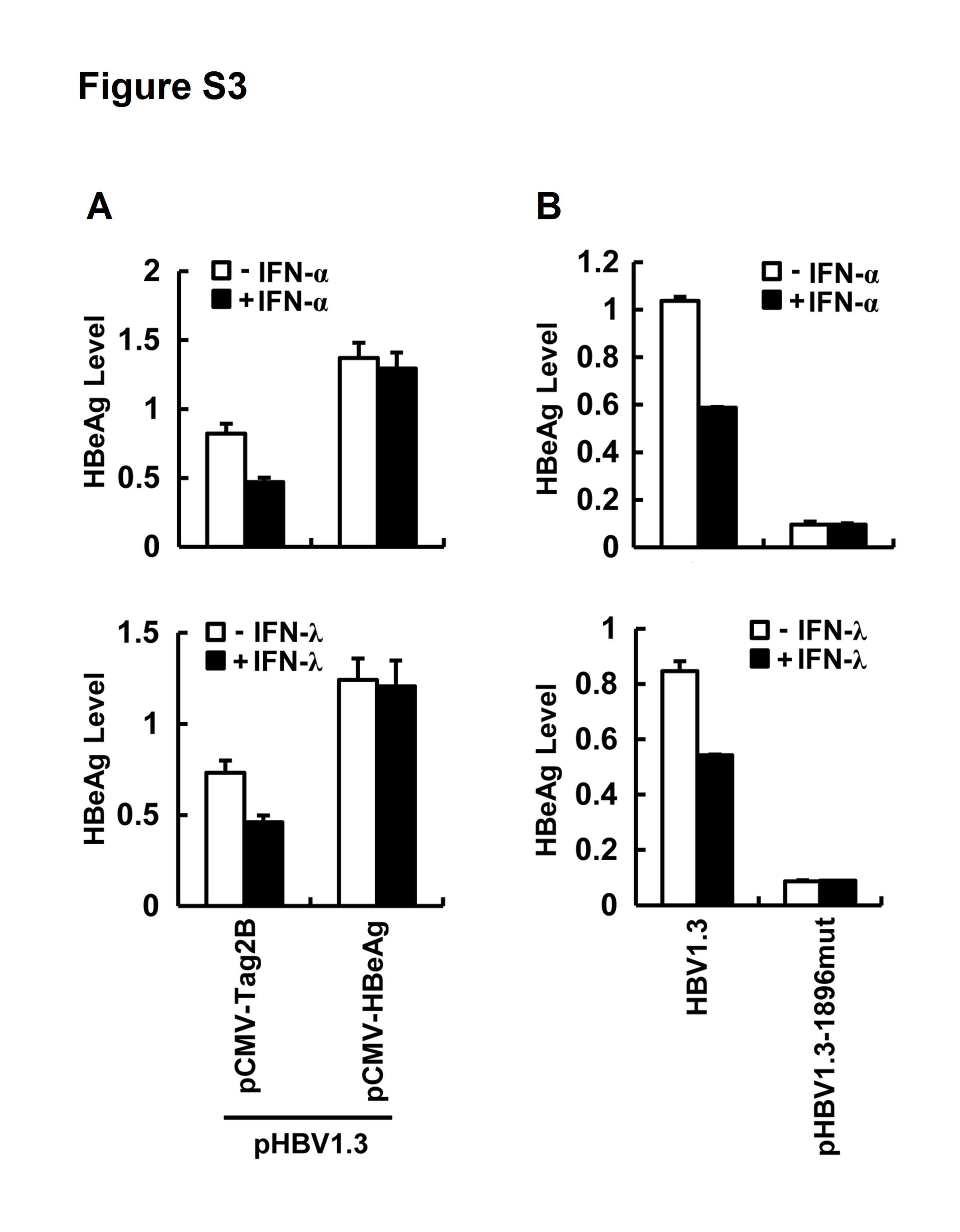


**Supplementary Figure S3. The impacts of HBeAg on the regulation of IFN-α and IFN-λ1 induced ISG expression and antiviral activities.**

(**A**) HepG2 cells were co-transfected with pHBV1.3 and pCMV-Tag2B or pCMV-HBeAg for 24 h and then treated with rhIFN-α (upper panels) or rhIFN-λ1 (lower panels) for another 24 h. Cells were harvested and lysed, and the levels of HBeAg in culture supernatants were measured by ELISA. (**B**) Huh7 cells were transfected with pHBV1.3 or pHBV1.3-1896mut for 24 h, and then treated with rhIFN-α (upper panels) or rhIFN-λ1 (lower panels) for another 24 h. Cells were harvested and lysed, and the levels of HBeAg in culture supernatants were measured by ELISA.

**
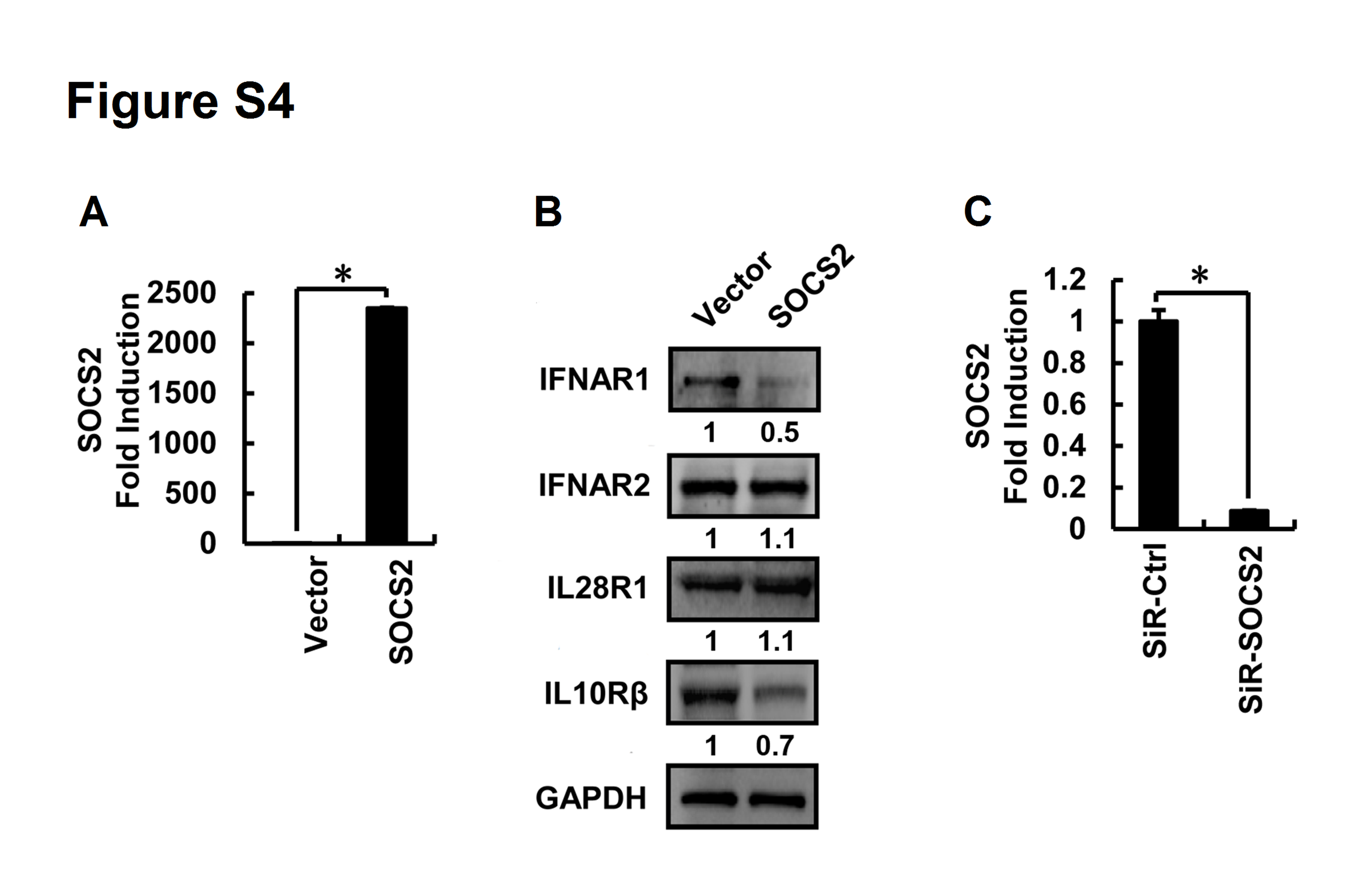
**

**Supplementary Figure S4. The function of SOCS2 in the regulation of IFN receptors expression.**

(**A**) HepG2 cells were transfected with pcDNA3.1 or pcDNA3.1-SOCS2 for 48 h. Cells were harvested and total RNA was extracted. The levels of SOCS2 mRNA were measured by real-time PCR. Data shown were means ± SE; n = 3. *p < 0.05. (**B**) HepG2 cells were transfected with pcDNA3.1 or pcDNA3.1-SOCS2 for 48 h. Cells were harvested and lysed, and IFNAR1, IFNAR2, IL-28R1 and IL-10Rβ proteins in the cell lysates were detected by Western blot analyses. (**C**) HepG2 cells were transfected with siRNA-Ctrl or siRNA-SOCS2 for 48 h. Cells were harvested and total RNA was extracted. The levels of SOCS2 mRNA were measured by real-time PCR. Data shown were means ± SE; n = 3. *p < 0.05.
